# Reliability of Diagnosis and Clinical Efficacy of Cranial Osteopathy: A Systematic Review

**DOI:** 10.1371/journal.pone.0167823

**Published:** 2016-12-09

**Authors:** Albin Guillaud, Nelly Darbois, Richard Monvoisin, Nicolas Pinsault

**Affiliations:** 1 CORTECS team, Univ. Grenoble-Alpes, Grenoble, France; 2 ThEMAS team, TIMC-IMAG laboratory, UMR CNRS-UGA 5525, Grenoble, France; 3 School of Physiotherapy, Grenoble-Alpes University Hospital, Grenoble, France; 4 Critical Thinking Research Federation, Univ. Grenoble-Alpes, FED 4270, Grenoble, France; University of Bern, SWITZERLAND

## Abstract

**Context:**

In 2010, the World Health Organization released benchmarks for training in osteopathy in which they considered cranial osteopathy as an important osteopathic skill. However, the evidence supporting the reliability of diagnosis and the efficacy of treatment in this field appears scientifically weak and inconsistent.

**Objectives:**

To identify and critically evaluate the scientific literature dealing with the reliability of diagnosis and the clinical efficacy of techniques and therapeutic strategies used in cranial osteopathy.

**Methods:**

Relevant keywords were used to search the electronic databases MEDLINE, PEDro, OSTMED.DR, Cochrane Library, and in Google Scholar, Journal of American Osteopathy Association and International Journal of Osteopathic Medicine websites. Searches were conducted up to end June 2016 with no date restriction as to when the studies were completed. As a complementary approach we explored the bibliography of included articles and consulted available previous reviews dealing with this topic.

**Study selection:**

Regarding diagnostic processes in cranial osteopathy, we analyzed studies that compared the results obtained by at least two examiners or by the same examiner on at least two occasions. For efficacy studies, only randomized-controlled-trials or crossover-studies were eligible. We excluded articles that were not in English or French, and for which the full-text version was not openly available. We also excluded studies with unsuitable study design, in which there was no clear indication of the use of techniques or therapeutic strategies concerning the cranial field, looked at combined treatments, used a non-human examiner and subjects or used healthy subjects for efficacy studies. There was no restriction regarding the type of disease.

**Search Results:**

In our electronic search we found 1280 references concerning reliability of diagnosis studies plus four references via our complementary strategy. Based on the title 18 articles were selected for analysis. Nine were retained after applying our exclusion criteria. Regarding efficacy, we extracted 556 references from the databases plus 14 references through our complementary strategy. Based on the title 46 articles were selected. Thirty two articles were not retained on the grounds of our exclusion criteria.

**Data extraction and analysis:**

Risk of bias in reliability studies was assessed using a modified version of the quality appraisal tool for studies of diagnostic reliability. The methodological quality of the efficacy studies was assessed using the Cochrane risk of bias tool. Two screeners conducted these analyses.

**Results:**

For reliability studies, our analysis leads us to conclude that the diagnostic procedures used in cranial osteopathy are unreliable in many ways. For efficacy studies, the Cochrane risk of bias tool we used shows that 2 studies had a high risk of bias, 9 were rated as having major doubt regarding risk of bias and 3 had a low risk of bias. In the 3 studies with a low risk of bias alternative interpretations of the results, such as a non-specific effect of treatment, were not considered.

**Conclusion:**

Our results demonstrate, consistently with those of previous reviews, that methodologically strong evidence on the reliability of diagnostic procedures and the efficacy of techniques and therapeutic strategies in cranial osteopathy is almost non-existent.

## Introduction

Osteopathy as a discipline was founded in the USA in 1874 by Andrew Taylor Still [1]. For the World Health Organization (WHO) osteopathy relies on manual contact for diagnosis and treatment, replacing the definition initially proposed by the World Osteopathic Health Organization. There exists a large heterogeneity in recognition and regulation of the practice of osteopathy across different countries, sometimes depending on whether practitioners are admitted to the medical community or not [2]. After the establishment of the first independent school of osteopathy in 1892, some graduates began to develop and teach new concepts in osteopathy. One of these concepts was cranial osteopathy, or “osteopathy in the cranial field”, elaborated by William Garner Sutherland in the early 20^th^ century. The biological model called upon to maintain cranial osteopathy is the disputed “primary respiratory mechanism”. Initially developed by Sutherland, this mechanism suppose that intrinsic rhythmic movements of the brain cause rhythmic fluctuations of cerebrospinal fluid and specific changes among dural membranes, cranial bones and the sacrum, that can be detected by palpation. In brief, cranial osteopathy consists of a non-invasive hands-on gentle manipulation of the skull to modify the parameters of this mechanism.

Objective data about the number of practitioners trained in cranial osteopathy or the frequency of use of cranial techniques in osteopathic practices are rare and inconsistent, mainly because of the lack of representativeness of the samples surveyed. Reports on the numbers of patients receiving cranial osteopathy vary widely, from 3.4% [3] to 94.8% [4] of those resorting to osteopathy. While some countries specifically prohibit teaching of cranial techniques (such as France [5]), nevertheless the WHO included cranial osteopathy among its benchmarks for training in osteopathy [2]. Such benchmarks require evidence based proof of safety, efficacy and quality assurance before a discipline can be introduced in the health care system. To achieve these criteria the diagnostic procedures have to be reliable and the proposed therapies to have been shown to be efficacious.

To date, three reviews of the literature (two systematic) have examined the intra and inter-examiner reliability of the diagnostic procedures used in cranial osteopathy [6–8]. However, all three had several limitations. That of Hartman *et al*. [7] cannot be considered as systematic, Green *et al*. [6] did not perform a systematic data analysis (i.e. they used a systematic method to extract relevant articles but described no standardized reliable method used to analyse the data), and Fadipe *et al*. [8], used a systematic method of analysis, the *quality appraisal tool for studies of diagnostic reliability* (QUAREL), but did not examine bias introduced by unblinded studies.

To our knowledge, four systematic literature reviews have been performed on the efficacy of therapeutic strategies in cranial osteopathy. Their qualities are variable; for example, the review conducted by Green et al. [6] has very broad inclusion criteria, with non-randomized or non-controlled studies included. Even if these points are not problematic from the standpoint of a general review (such as proposed by Green et al.), reviews that draw conclusions concerning clinical efficacy (such as ours) should take into account the level of evidence. The reviews by Jäkel & von Hauenschild suffer either from non-systematic analysis of results [9] or unsuitable methods for the analysis of bias [10]. Finally, Ernst [11] uses more suitable methods for the determination of quality and an analysis of bias, and suggests eligibility criteria for studies that are in line with those conventionally used to assess efficacy.

Considering all these points, we conducted two systematic reviews to identify and critically evaluate the scientific literature dealing with 1) the reliability of the diagnostic process and 2) the clinical efficacy of techniques and therapeutic strategies used in cranial osteopathy.

## Methods

### Literature sources and search

In August 2015 we searched MEDLINE, PEDro, OSTMED.DR, and the Cochrane Library databases and as well as Google Scholar, the Journal of American Osteopathy Association (JAOA) and the International Journal of Osteopathic Medicine (IJOM) websites.

The search strategy was as follows:

- for reliability studies, we started with the combination of keywords [“reliability” OR “agreement” OR “reproducibility”] AND [“cranial” OR “*craniosacral*” OR “cranium” OR “*primary respiratory mechanism*”]. When the number of references exceeded 100 hits with the above equation, we added [“osteopathy” OR “osteopathic”].
- for efficacy studies, we used the combination of keywords [“cranial manipulation” OR “osteopathy in the cranial field” OR “cranial osteopathy” OR “craniosacral technique”] AND [“medicine” OR “treatment” OR “therapy” OR “technique” OR “manipulation” OR “osteopathy” OR “osteopathic”].

Depending on the interfaces, keywords were entered in a classic search bar or, when possible, by selecting an advanced search tool for titles, abstracts and keywords. We performed the search until 30 June 2016 without date limitation of publication (i.e. Date of publication filter in search criteria was not filled).

In order to be exhaustive, we conducted a second search using a complementary approach consisting of an analysis of the bibliography section of included articles, consultation of the available systematic reviews dealing with our topic, and contacts with study authors or professional institutions to identify additional studies.

### Eligibility criteria

#### Reliability of the diagnosis

We considered studies including a comparison of the results obtained by at least two examiners (inter-rater reliability) or the results of at least two examinations by the same examiner (intra-rater reliability). We only considered studies on humans (patients or healthy volunteers).

#### Efficacy studies

For efficacy studies, we included only randomized-controlled-trials (RCT) or crossover studies on patients, but not studies on healthy subjects.

Other exclusion criteria included articles not published in English or French, studies with non-RCT or non-crossover study design, studies in which there is no clear indication for the use of cranial osteopathy techniques and studies in which a combination of methods were proposed, those that used non-human simulators, and finally studies for which we could not obtain the full text version. We made no restriction in terms of the type of disease, healthcare services involved or health outcomes.

### Study selection

For inclusion in our review, studies had to meet the aforementioned eligibility criteria. For study selection, we considered all techniques claimed by the authors to belong to the field of cranial osteopathy or mentioned in the classical osteopathic literature. If in doubt, we considered the technique to be inside the field. Studies that described the use of techniques or diagnostic/therapeutic strategies from cranial osteopathy together with other diagnostic/therapeutic modes but without performing subgroup analysis were excluded.

The systematic selection process was composed of 3 steps. Firstly we made a selection by title. Duplications due to overlap in the coverage of the databases and off-topic studies were excluded. Secondly, the abstracts of each study were analyzed. Studies that did not meet the eligibility criteria on the basis of the content of their abstracts were excluded. Full-texts of the remaining studies were obtained and the eligibility criteria were again applied.

For references obtained with the complementary approach, the study abstracts were analyzed and, if required, the full-text versions obtained to determine whether the studies met our eligibility criteria.

### Data extraction

The data extracted included: study design (including randomization and blinding procedures), sample size and characteristics (such as age and/or disease or inclusion criteria), main outcomes and results obtained.

For reliability studies we added information regarding examiners (*e*.*g*., number, qualification, expertise) as well as the statistical methods used.

For efficacy studies, we added the primary outcome to be evaluated and a precise description of the treatments applied.

### Assessment of risk of bias

In accordance with the guidelines [12], study screening and risk of bias assessments (for reliability and efficacy) were done in duplicate by two screeners using standard forms. Disagreements between the two screeners were resolved by consensus.

### Assessment tool for reliability studies

For reliability studies, we assessed the risk of bias in each study using a modified version of the quality appraisal tool for studies of diagnostic reliability (QAREL) [13]. Briefly, QAREL is an 11-item checklist that covers 7 key domains: the spectrum of subjects; the spectrum of examiners; examiner blinding; effects of order of examinations; the suitability of the time-interval between repeated measurements; appropriate test application and interpretation; and appropriate statistical analysis. Our intention was to use QAREL to analyze only the methodological risk of bias. We considered items 1 (Was the test evaluated in a sample of subjects who were representative of those to whom the authors intended the results to be applied?) and 2 (Was the test performed by raters who were representative of those to whom the authors intended the results to be applied??) of the QAREL as not referring to risk of bias but to applicability of the results, defined by Atkins *et al*. [14] as the extent to which the effects observed in published studies are likely to reflect the expected results when a specific intervention is applied to the population of interest under ‘‘real-world” conditions. In the same context, the part of QAREL related to statistical analysis (items 10 and 11) were not used and we conducted a separate analysis and interpretation of the statistics used in the included studies. Note that our analysis was not so far from the QAREL items but benefited from more precise interpretation criteria, as detailed later in the text. Lastly, for remaining items of the QAREL, we did not consider the items n° 5 (Were raters blinded to the results of the reference standard for the variable being evaluated?) and n° 9 (Was the time interval between repeated measurements compatible with the stability of the variable being measured?) because there are no reference standards or evidence regarding the stability of outcomes in the field of cranial osteopathy. To recap we selected from the QAREL checklist items 3 (Were raters blinded to the findings of other raters during the study?), 4 (Were raters blinded to their own prior findings of the test under evaluation?), 6 (Were raters blinded to clinical information that was not intended to form part of the study design or testing procedure?), 7 (Were raters blinded to additional cues that are not part of the test?) and 8 (Was the order of examination varied?).

Two additional items were added to the previous checklist in order to cover parameters known to influence the reliability of procedures involving manual therapies: 1) the personal expertise of the examiner and 2) the existence of an appropriate blinding procedure for examiners when testing subjects simultaneously. In fact, the personal expertise of the examiner has been shown to strongly influence the reliability of testing procedures in the field of manual therapy (see [15], [16] or [17] for examples of reviews on muscle testing, spinal palpation or sacro-iliac joint tests, respectively). A suitable blinding procedure would be to have two examiners performing tests simultaneously (generally one to the feet, the other to the head).

### Rating rules for reliability studies

Each of our 7 items in a given study could be rated as having ‘Low’ or, ‘High’ risk of bias, or ‘Unclear’ risk of bias when the report was insufficiently detailed. For the personal expertise of the examiner, we rated this item with high risk of bias when examiners were students or had not completed their training in the discipline, with low risk of bias when examiners had graduated and an unclear risk of bias when this information was unavailable.

The overall evaluation for a study, corresponding to general assessment of bias item, was: ‘High risk’ of bias when at least one item was rated as high risk; ‘Major doubt’ as to the overall risk of bias when more than two items had an unclear risk of bias with all other items being low risk; ‘Minor doubt’ as to the overall risk of bias when two or less items was/were judged to have unclear risk of bias, with all others having low risk; and overall ‘Low risk’ of bias when all items were rated as having low risk of bias.

### Statistical analysis interpretation for reliability studies

Together with the general appraisal of bias, we analyzed and interpreted the statistical analysis of results before concluding. Drawing inspiration from the QAREL items for statistical analysis we tried to be more precise in our interpretation criteria. In fact, we considered reliability or agreement as being satisfactory when classified, respectively, as excellent according to the Fleiss’ classification (*i*.*e*. with an intraclass correlation coefficient (ICC) above 0.75) or as almost perfect according to the Landis & Koch classification (*i*.*e*. with a kappa coefficient (κ) above 0.81) [18,19]. The targets we set for an acceptable standard might be considered as very high for techniques in the manual therapy field but, considering that cranial osteopathy is founded on a disputed concept (the primary respiratory mechanism), in our opinion this statistical precaution appears to be necessary.

As far as statistical methods are concerned we considered, in line with Lucas et al. [13], that intraclass correlation was appropriate for assessing inter-rater reliability on quantitative, ordinal, interval, and ratio variables, while kappa is a useful measure of inter-rater reliability for nominal (i.e., categorical) variables. To be precise, ICC assesses rating reliability by comparing the variability of different ratings of a given subject to the total variation across all ratings and all subjects. Thus, ICC is suitable for studies with two or more raters, and may be used when all subjects in a study are rated by multiple raters, or when only a subset of subjects is evaluated by multiple raters and the rest are rated by only one. In other words, ICC is a useful estimate of reliability because it is highly flexible. Other correlation statistics, such as Spearman or Pearson analyses, percentage agreement or measures of precision (such as confidence limits) are not appropriate for estimating reliability [13,20].

#### Assessment tool for efficacy studies

In order to assess the risk of bias in efficacy studies we used the Cochrane risk of bias tool [21]. In short, the Cochrane risk of bias tool estimates the risk of bias arising from six domains: generation of the allocation sequence, concealment of the allocation sequence, blinding, incomplete outcome data, selective outcome reporting, and other biases.

#### Rating rules for efficacy studies

The Cochrane risk of bias tool allocates a level of risk by domain, evaluated as ‘Low’ or ‘High’ risk of bias, or ‘Unclear risk’ of bias when the information given was insufficient. To determine this last point the 2010 CONSORT checklist was consulted [22]. In fact this checklist, together with the explanatory and elaboration document provided by CONSORT, provides detailed information to evaluate items of the Cochrane risk of bias tool. We can reasonably consider that if the information available on the study did not enable us to complete the checklist, an “unclear” risk of bias should be allocated to the item. Concerning the last item of the Cochrane risk of bias tool, that of “other biases”, our strategy was to search any potential source of bias typical of clinical trials, such as absence of placebo treatment, compliance bias etc. (see [23] for an inventory). This states that such biases should be of sufficient magnitude to have a notable impact on the results or conclusions of the trial, whilst recognizing that subjectivity is involved in any such assessment.

However, considering that a high risk of bias in the domain of blinding is inherent to the field of manual therapies, we modified the overall risk of bias measurement. Thus for studies in the field of manual therapy the overall risk of bias would be: ‘High’ when at least one item in addition to of “blinding” had a high risk of bias; ‘Major doubt’ regarding the risk of bias when two or more items had an unclear risk of bias, with all other domains (aside from blinding) having a low risk of bias; ‘Minor doubt’ regarding the risk of bias when only one item was judged to have an unclear risk of bias, with all others (aside from blinding) having a low risk of bias; and ‘Low risk’ of bias when all items other than blinding had a low risk of bias.

All studies included in our review were analyzed using this last procedure.

## Results

### Reliability studies

**Fig 1:**
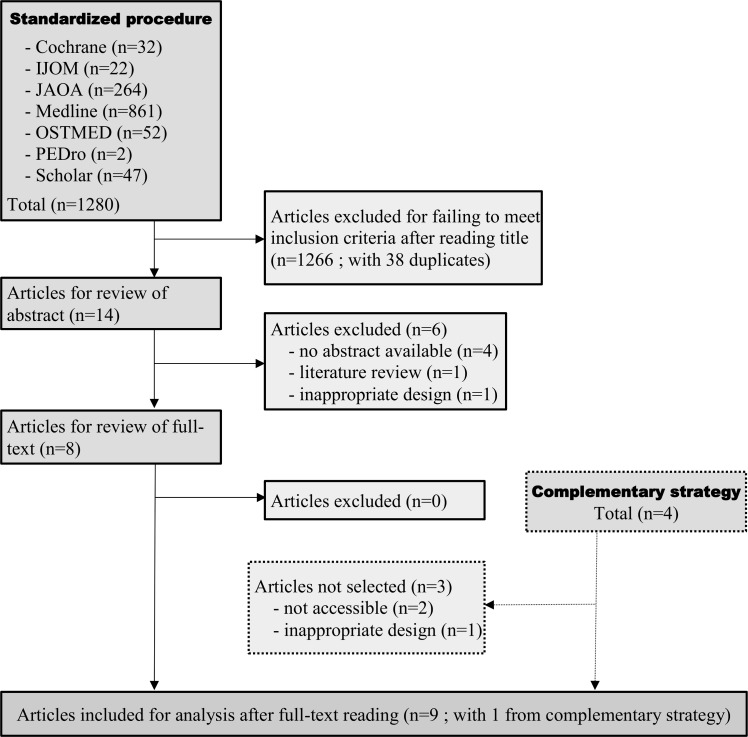
Flow chart of the study selection process for the systematic review of studies dealing with the reliability of diagnosis in the field of cranial osteopathy.

Our standard search procedure identified 1280 articles, of which eight met the inclusion criteria (Fig 1). Our complementary search strategy gave four more articles with only one meeting our inclusion criteria. Details of these studies are summarized in Table 1.

**Table 1:** Summary of included studies dealing with the reliability of diagnosis in cranial osteopathy.

First authors	Subjects (number; disease status; age in yrs)	Raters (number ; degree(s) ; expertise)	Study Characteristics & Parameter(s)	Reliability Measure Used	Main Results
Upledger [24]	N = 25 ; not reported; A = 3–5	N = 4 ; one DO (founder of CST) and three MDs; one trained by the CST founder and two considered as “skilled examiners”	Inter-examiner : (1) CRI-F; (2) restriction of motion in several areas (19 modalities)	Reliability coefficient (no more information)	**inter**: (1) : missing data; (2) : coefficient ranged from 0 to 1 for all modalities and examiners
Wirth-Pattullo [25]	N = 12; history of trauma, surgery, or “learning disabilities” ; A = 10–62	N = 3 (X, Y and Z) ; PT trained in CST ; 2–4 yrs	Inter-rater : cranial motion F	ICC (2,1)	**inter**: X-Y: -0.33 ; X-Z: -0.,60 ; ;Y-Z: 0.49 ; X-Y-Z: -0.2
Norton [26]	N = 9 ; healthy ; A = 22–28	N = 6 ; MD-DO ; “extensive training and experience in cranial osteopathy”	• intra-rater of : (1)flexion-duration of the CR ; and (2)duration of cranial cycles (second) • inter-rater : CR-F (cpm)	Pearson product-moment correlation coefficient	• **intra : **(1) : missing data; (2) • **inter :* ***-0.32 to -0.28
Hanten [27]	N = 30 ; any disease or trauma about the skull or spine ; A = 22–54	N = 2 (X & Y) ; PT students ; 11 months	Intra & inter-rater : CR-F	ICC (1,1)	• **intra :** X : 0.78 ; Y : 0.83 • **inter : **0.22
Rogers [28]	N = 28 ; healthy ; A = 18–48	N = 2 (X & Y) ; one PT & one RN trained in CST ; : 5 & 17 yrs respectively	Intra & inter-rater : CR-F to the head and feet	ICC (2,1)	• **intra :** X : 0.18 for head and 0.30 for feet ; Y : 0.27 for head and 0.29 for feet • **inter** : 0.08 (head) and0.19 (feet)
Vivian [29]	N = 48 ; not reported; some subjects could have chronic or recurrent pain ; A = 7–63	N = 2 ; DO ; 12 & 15 yrs	Inter-rater of : (1) presence of a partially flexion-restricted motion of the skull ; (2) presence of a total flexion-restriction motion of the skull	Cohens's kappa	**inter :** (1) : -0.02 ; (2) : -0.09.
Moran [30]	N = 11 ; healthy ; A = 18–44	N = 2 ("X” & “Y”) ; DO ; 4.5 & 6.5 yrs	Intra & inter-rater of : CRI-F to the head and/or sacrum	ICC (2,1)	• **intra** X : 0.65 for sacrum; 0.47 for head; Y : 0.52 for sacrum; 0.73 for head • **inter : **0.0 for X and Y to the head and 0.05 for X and Y to the sacrum
Sommerfeld [31]	N = 49 ; healthy ; A = 19–61	N = 2 ; DO ; 7 yrs	Intra & inter-rater of : PRM-frequency ; PRM flexion-stage duration ; ratio of the flexion-stage and the extension-stage duration of the PRM	95 % limit of agreement (Visual representation)	**intra & interexaminer** agreement could not be described beyond chance agreement.
Halma [32]	N = 48 ; 16 asthma, 17 headache, 15 healthy ; A : 18–75	N = 2 ; MD-DO ; 14 & 6 yrs	Intra-rater of : (1) CRI-F ; (2) cranial strain patterns ; (3) quadrants of restriction with 4 modalities	Cohens's kappa with 95 % confidence intervals	**intra** (1) : 0.23 ; (2) : 0.67; (3) : from 0.33 to 0.52 according to modalities

Note: "N": number; "A": age; "DO": doctor of osteopathy; "PT": physical therapist; "RN": registered nurse; "CST": craniosacral therapy; "CRI": cranial rhythmic impulse; "F": frequency; "CR": cranial rhythm; "R": rater; "PRM": primary respiratory mechanism; "ICC": intraclass correlation coefficient

For two articles our analysis led us to consider their results as unusable. We considered as unusable results that could not be interpreted because of serious mistakes in data presentation or calculation, aside from the meaning of the results in terms of reliability. In fact, as previously noted by Hartman & Norton [7], the article by Upledger [24] showed many serious biases such as selective reporting, misreporting, miscalculation *etc*. Moreover, the statistical methods used to demonstrate reliability were inappropriate. For the study of Sommerfeld *et al*. [31], the main problem was the absence of a Bland & Altman graph (or data allowing it to be built) whereas the authors clearly stated in their methods of statistical analysis that this approach was used.

**Fig 2:**
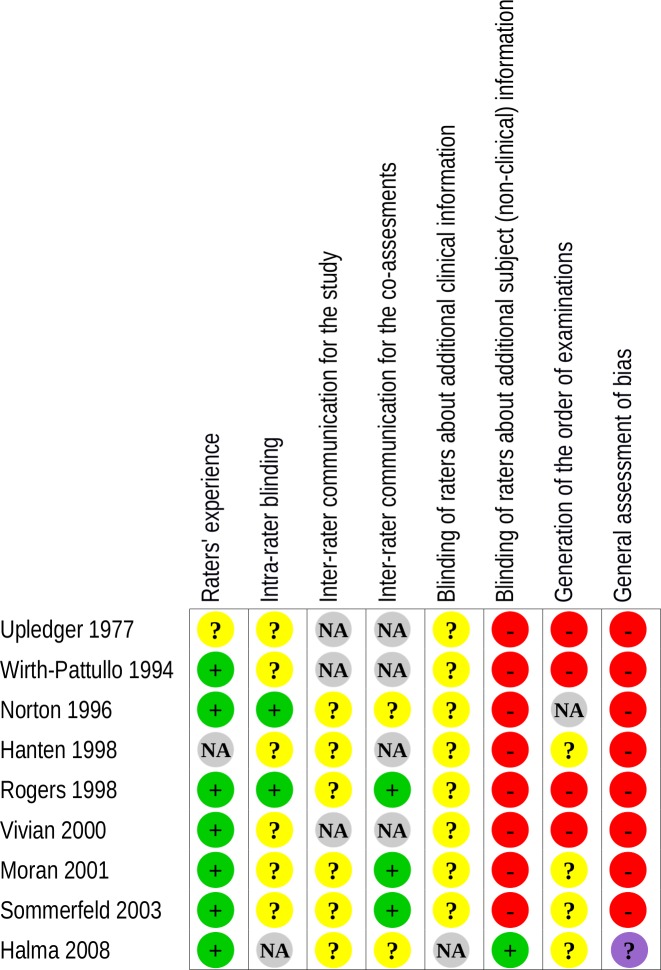
Assessment of methodological risk of bias for each of the included reliability studies. Green indicates a low risk of bias, yellow an unclear risk of bias and red a high risk. Grey indicates non-applicable items. For the overall assessment of bias, purple indicates major doubt as to the overall risk of bias.

**Fig 3:**
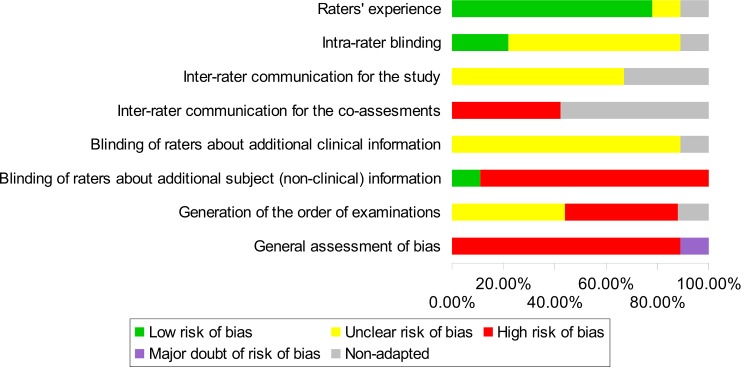
Assessment of methodological risk of bias for the reliability studies taken together. Green indicates a low risk of bias, yellow an unclear risk of bias and red a high risk. Grey indicates non-applicable items. For the overall assessment of bias, purple indicates major doubt as to the overall risk of bias.

Critical appraisal led us to conclude that we had a major doubt for the general risk of bias of one study [32] and that all other reliability studies included in our review demonstrated a high risk of bias, particularly due to a lack of blinding of the examiners (Figs 2 and 3).

### Efficacy studies

**Fig 4:**
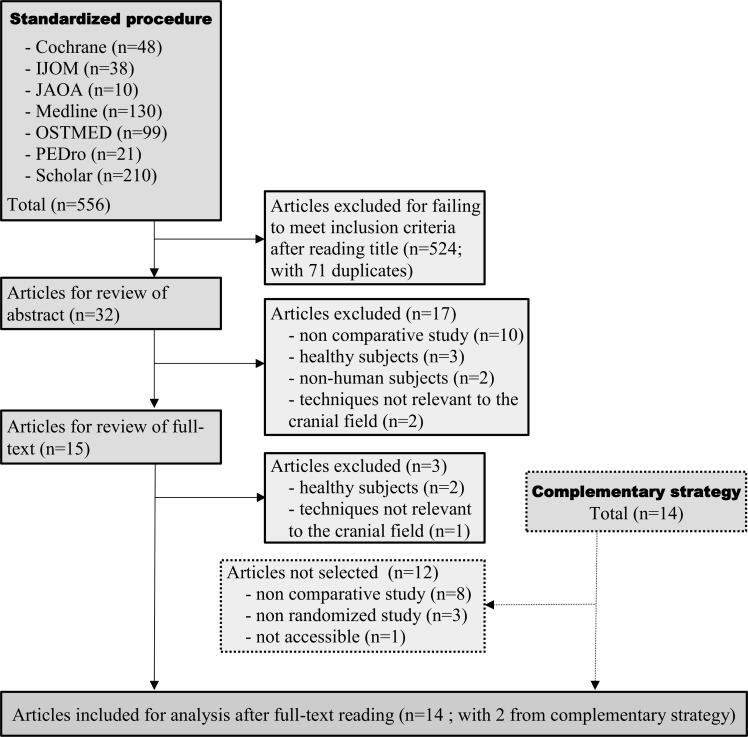
Selection process for studies dealing with the clinical efficacy of techniques and therapeutic strategies used in cranial osteopathy.

Our standardized search procedure identified 556 articles, of which 12 met the inclusion criteria (Fig 4). Our complementary search strategy found 14 more articles with 2 reaching our inclusion criteria. Details of these fourteen studies are summarized in Table 2.

**Table 2:** Description of included studies dealing with the clinical efficacy of techniques used in osteopathy in the cranial field.

	First author	Disease & number of participants	Intervention and comparison	*Primary study outcome* & result	*Other outcomes* & results
**Low risk of bias**	Elden [42]	Pelvic Girdle Pain: 123	EG “standard treatment + craniosacral therapy” / “standard treatment” G	*Sick leaves & pain intensity in the morning and the evening*. Results showSSD but no MCID for pain intensity in the morning in favour of EG (VAS increased by 7mm in CG and decreased by 0.5mm in EG after treatment)	None
	Haller [44]	Non specific neck pain: 54	EG “craniosacral therapy protocol” / PG “sham of the craniosacral therapy protocol”	*Pain intensity*.Results showSSD & MCID 3 months after treatment for the pain intensity in favour of EG (-21 and -16.8mm after 8 and 20 weeks, respectively)	• *32 criteria (16 outcomes immediately after treatment and at 3 months)*. 1. Pain on movement *(Pain on Movement Questionnaire)*. 2. Maximum pressure pain sensitivity : point of maximum pain, levator scapulae, trapezius and semispinalis capitis muscles (algometer). 3. Functional disability (*Neck Disability Index*). 4. Quality of life (through two subscales of the *12-item Short Form Health Survey*). 5. Well-being (*16-item Questionnaire for Assessing Subjective Physical Well-being*). 6. Anxiety and depression (*Hospital Anxiety and Depression Scale*). 7. Stress perception (*Perceived Stress Questionnaire*). 8. Pain acceptance (through the subscale “Positive life constrution Scale” of the *Emotional/Rational Disease Acceptance Questionnaire*). 9. Body connection (through two subscales “body awareness” and “body dissociation” of the *Scale of Body Connection*). 10. Global impression of improvement (*Patients’ ratings of their Globa Impression of Improvement*). • Results show SSD to 7 outcomes after treatment and 5 outcomes at three months in favor of the EG group**.
	Castro-Sànchez [45]	Non specific low back pain: 64	EG “craniosacral therapy”/ CG “Classic massage”	*Roland Morris Disability Questionnaire*. Results show no SSD	• *32 criteria (16 outcomes immediately after treatment and one month later)*. 1. Low back pain disability (*Oswestry Low Back Pain Disability Index*). 2. Pain intensity (*10-point numeric pain rating scale*). 3. Kinesiophobia (*Tampa Scale of Kinesiophobia*). 4. Hemoglobin oxygen saturation, systolic blood pressure, diastolic blood pressure and hemodynamic (cardiac index) (*Electro Interstitial Scanner*). 5. Interstitial levels of sodium, serum potassium, chloride, phosphate, ionized or free calcium, magnesium and lactic acid (*Electro Interstitial Scanner*). 6. Isometric endurance of trunk flexor muscles (*McQuade test*). 7. Lumbar mobility in flexion (finger-to-floor distance). • Results show SSD to 6 outcomes after treatment and 3 outcomes one month later in favour of CST group**.
**Major doubt on risk of bias**	Hanten [33]	Tension-type headache : 60	EG “CV-4 technique as described by Upledger and Vredevoodg”/ untreated G(1)/ resting position G(2)	None	*2 criteria (VAS for Pain intensity and Pain affect) immediately after treatment*. Results showdifference between pretest and post-test for Pain intensity [untreated G : 7.8 mm ; resting position G : 11.2 mm; EG : 19.3 mm] and Pain affect [untreated G : 2.9 mm; resting position G : 7.6 mm ; EG : 14.9 mm]
	Hayden [34]	Infantile colic : 28	EG “standard cranial osteopathic techniques” */* untreated G	None	*3 criteria immediately after treatment*, *that are Crying and sleeping daily durations (parent reporting) andDuration of parent holding and rocking (parent reporting)*. Results show SSD for all criteria (daily amount of crying : −1h, sleeping time : −1.17h and helding or rocking time : −0.7h) after treatment in favor of the EG.
	Nourbakhsh [36]	Lateral epicondylitis : 23	EG “The OEMT [Oscillating-energy Manual Therapy] (V-spread) was administered based on the standard method described in many osteopathic texts.”/ PG	None	• *7 criteria (4 outcomes immediately after treatment and 3 outcomes at 6 months)*. 1. Grip strength (Jamar dynamometer). 2. Functional level (*Patient-Specific Functional Scale*). 3. Pain intensity (11-point scale). 4. Pain limited activity (11-point scale). Results show differences immediately after treatment for grip strength (PG: -1.9; EG: +12.3), functional level (PG: +4.7; EG: +14.5), pain intensity (PG: -0.5; EG: -3.1) and pain limited activity (PG: -0.1; EG: +3.3) and no SSD at 6 months for functional level, pain intensity and pain limited activity.
	Sandhouse [37]	Myopia & hyperopia : 29	EG “The specific OMT technique performed was balanced membranous tension”/ PG	None	• *12 criteria immediately after treament*. 1. Presence of a cranial dysfunction (manual evaluation). 2. Visual acuity (right and left) (distance visual acuity testing). 3. Accomodation amplitude (right and left) (Donder push-up testing). 4. Stereoscopic visual acuity (local stereoacuity testing). 5. Pupillary size (right and letft ; under bright light and dim light) (Pupillary testing). 6. Ocular deviation (Cover test with prism neutralization). 7. Near point of convergence (break point and record point) (distance in cm). Results show SDD for 1 criterion out of 12 between EG and PG : the left pupillary size measured under bright light with respectively +0.13mm and -0.40mm of difference between after and before intervention for EG and PG, respectively.
	Castro-Sánchez [38]	Fibromyalgia : 92	EG “a craniosacral therapy protocol”/ PG	None	• *75 criteria (25 outcomes immediately after treatment and 2 months and 1 year later)*. 1. Body composition (extracellular, cellular and lean mass analysed with bioelectrical impendance). 2. Pain at 18 tender point sites (pressure algometer). 3. Heart rate, temporal standard deviation of RR segments (HRV) and root mean square deviation of HRV index (Holter). 4. Clinical global impression of improvement (7-level Likert scale). • Results : number of criteria above 20*.
	Matarán-Peñarrocha [39]	Fibromyalgia : 104	EG “a craniosacral therapy protocol”/ PG	None	• *54 criteria (18 outcomes immediately after treatment*, *6 months and 1 year later)*. 1. Pain intensity (VAS). 2. Quality of life (SF-36 : one outcome for each of the 8 questionnaire sections − thus 8 outcomes). 3. Sleep quality (*Pittsburgh Sleep Quality Index* : one outcome for each of the 6 questionnaire sections − thus 6 outcomes). 4. Depression state (*Beck depression inventory*). 5. Trait anxiety and state anxiety (2 outcomes) (*State Trait Anxiety Inventory*). • Results : number of criteria above 20*.
	Amrovabady [40]	Attention deficit hyperactivity disorder : 24	EG “Craniosacral therapy”/ standard treatment G	None	*10 criteria immediately after treatment[the Conners Parents Rating Scale* 48-question version (divided into 5 sub-outcomes and the *Child Symptoms Inventory-4th* (divided into 5 sub-outcomes)]. Results show SSD for all results in favor of the EG with, for instance, a total CPRS difference of +0.58 in the standard treatment G *vs*. +7.5 in the EG.
	Árnadóttir [41]	Migraine : 20	Cross-over range on 12 wks with 2 G(“Upledger Craniosacral therapy” *vs*. no treatment)	*HIT-6 questionnaire*. Results show SSD immediately after treatment (effect size : 0.48) and 1 mo after treatment (effect size : 0.43).	None
	Bialoszewski [43]	Non specific low back pain : 55	EG “Craniosacral therapy”/ trigger point therapy G	None	• *8 criteria immediately after treatment*. 1. Pain severity (VAS). 2. Pain intensity (modified Laitinen questionnaire). 3. Pain frequency (modified Laitinen questionnaire). 4. Analgesic use (modified Laitinen questionnaire). 5. Functional impact of pain (modified Laitinen questionnaire). 6. Lombosacral mobility (Schober test). 7. Resting tension of the multifidus muscle (right and left) (Electromyography). Results show no SSD after treatment for all criteria.
**High risk of bias**	Mehl-Madrona [35]	Chronic asthma : 89	CST G “standard craniosacral therapy treatments in accordance with the protocol taught at the Upledger Institute in Michigan”/ acu G / CST + acu G/ PG/ waiting list	None	• *21 criteria (7 outcomes at 2 weeks*, *2 months and 6 months after treatment*). 1. Pulmonary function (no more information). 2. Quality of life *(Asthma Quality of Life* and *SF-36)*. 3. Mood state (*Profile of Mood State*). 4. Depression state *(Beck Depression Inventory)*. 5. Medication use over the past year (dose and frequency). • Results : Selective reporting of results. Number of criteria above 20*.
	Raith [46]	Preterm infants : 30	EG “standardised craniosacral therapy”/ standard treatment G	*General Movement Assessment*. Results show no SSD.	*1 criterion immediately after treatment (General Movement Optimality Score)*.Results show no SSD.

*Legend*. ***EG***: *experimental group;* ***G***: *group;* ***SSD***: *significant statistic difference*; **CST**: *craniosacral therapy*; **acu**: *acupuncture*; **PG**: *placebo group*; **VAS**: *visual analogic scale*; ***MCID***: *minimal clinically important difference*.

*Considering the risk of inflated alpha value and for sake of clarity, the results of the studies that both had not chosen primary study outcomes and had used more than 20 criteria were not reported.

** No detail is given for sake of clarity.

**Fig 5:**
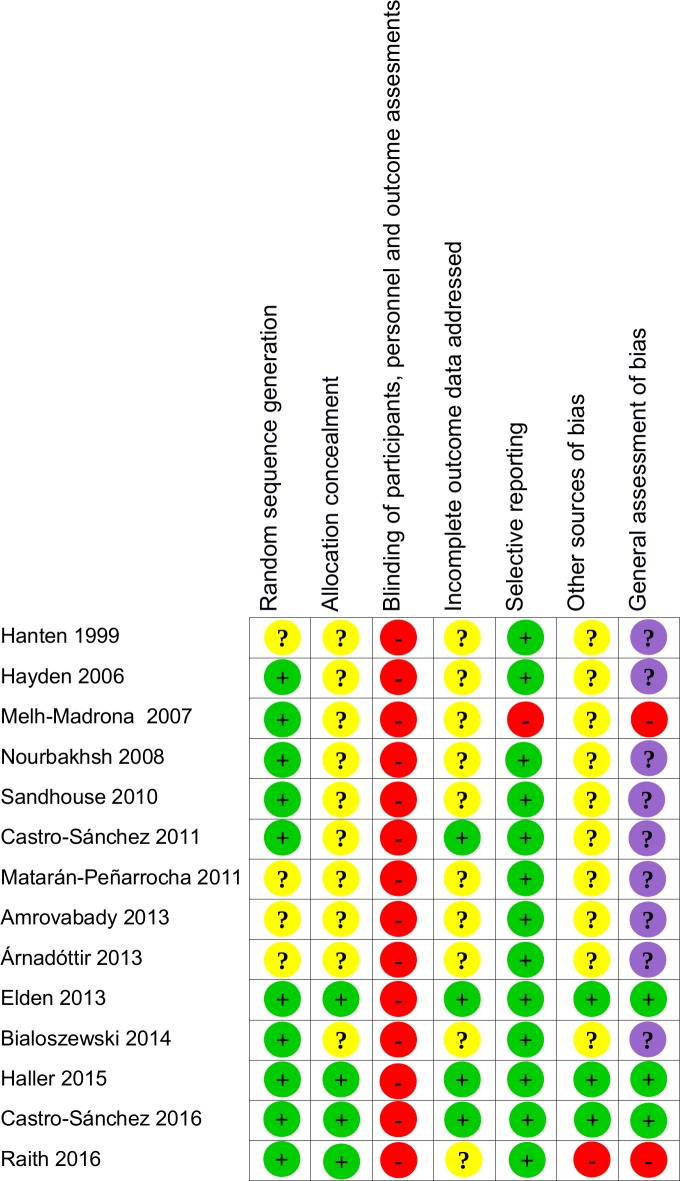
Assessment of methodological risk of bias for each efficacy study included. Green indicates a low risk of bias, yellow an unclear risk of bias and red a high risk. Grey indicates non-applicable items. For the general assessment of bias, purple shading indicates a major doubt as to the overall risk of bias.

**Fig 6:**
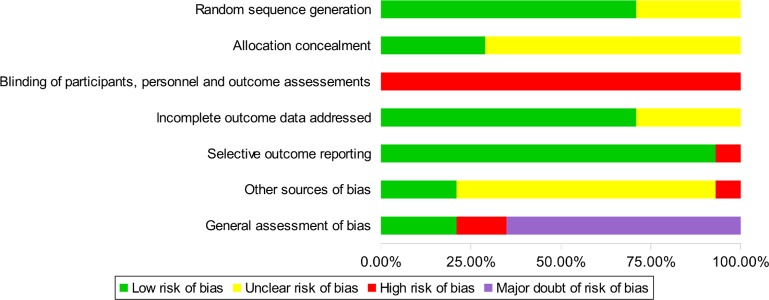
Assessment of methodological risk of bias for the efficacy studies taken together. Green shading indicates a low risk of bias, yellow an unclear risk of bias and red a high risk. Grey shading colour indicates non-applicable items. For the general assessment of bias, purple shading indicates a major doubt as to the overall risk of bias.

Among the included studies 2 were found to have a high risk of bias [35,46]; for 9 there was major doubt regarding the risk of bias [33,34,36–41,43] and 3 were evaluated as having a low risk of bias [42,44,45] (Figs 5 and 6). The principle sources of bias found in studies were the absence of a principal evaluation criterion, lack of correction method for inflated alpha values, no interpretation of the clinical relevance of the results, lack of comparability between proposed treatments and subjective evaluation with an unclear or non-existent blinding method

## Discussion

In this review we aimed to identify and critically evaluate the scientific literature dealing with 1) the reliability of the diagnostic process and 2) the clinical efficacy of techniques and therapeutic strategies used in cranial osteopathy.

Concerning the diagnostic processes, we found 9 studies that met our inclusion criteria [24–32]. Eight of them demonstrated a high risk of bias and we had a major doubt regarding the risk of bias for the other one [32]. Note, that this last study reported unreliable results in terms of our criteria. Eight studies addressed the issue of inter-rater reliability [24–31] and 6 addressed the issue of intra-rater reliability [26–28,30–32]. Whether for inter- or intra-rater reliability studies, results were either unusable or did not show reliability for any of the investigated parameters.

Regarding the efficacy of techniques used in cranial osteopathy, our review shows that for 14 studies meeting our inclusion criteria, only three had a low risk of bias [42,44,45], for nine there was major doubt regarding the risk of bias [33,34,36–41,43] and two were rated with high risk of bias [35,46]. While this may be open to debate, we only considered as evidence those studies with low risk of bias. The three studies fulfilling these criteria are discussed below.

First, the study by Elden et al. [42] was a randomized multicenter single blind controlled trial designed to investigate the efficacy of craniosacral therapy as a complement to standard treatment, compared with standard treatment alone, for pelvic girdle pain during pregnancy. The three main outcomes were clearly identified, precise and clinically relevant, although pain was a subjective outcome as it is self-reported by the patient. However, many secondary outcomes were assessed by the study but the statistical analysis did not propose any correction method for inflated alpha values due to multiple analyses. Moreover, the results show a significant statistical difference immediately after the intervention for only one of the three main outcomes, which is pain in the morning, and three of the 17 secondary outcomes. However, we have to mention that the modification of the pain in the morning, even if statistically significant, is mainly due to increased pain in the control group than to a decrease in the intervention group. Considering that Elden et al. proposed a sample size calculation before the study start, we can reasonably consider the lack of statistical significance for other outcomes as not being due to insufficient statistical power. Lastly, we note that there was almost no contact with the practitioner in the standard treatment group of the study. This methodological point induces a confusion between the specific effect of the techniques used and their non-specific effects, making the results hard to interpret, In fact, the lack of contact with a practitioner in the standard group (particularly when subjective outcomes such as VAS are used) leads to many contextual effects including, but not limited to, the individual practitioner and patient’s belief [47], the doctor–patient relationships [48,49] or the clinicians expectation [50]. Together with the other limitations, this point led us to conclude that this study does not contribute to the body of evidence for the specific efficacy of the techniques used, but could suggest contextual effects of the treatment.

The second study rated as having low risk of bias, by Haller et al. [44] aimed at investigating craniosacral therapy (CST) compared to sham treatment in patients with chronic non-specific neck pain. The primary outcome was pain intensity assessed with visual analog scale and 16 secondary outcomes were investigated. Data (between CST and sham groups) were compared immediately and three months after the intervention. The results showed statistical and clinically relevant differences in favor of CST for the primary outcome and seven of the secondary outcomes immediately after treatment. At three months the results remained statistically and clinically relevant for the primary outcome and statistical differences still existed for five of the secondary outcomes. While this study is methodologically relatively strong, it nevertheless has some limitations. As for the study by Elden et al. [42] the main outcome is patient self-reported pain and no correction method for inflated alpha values is proposed despite the numerous analyses reported. Moreover, we note that three practitioners intervened in the CST arm and only one in the sham arm. Considering the importance of the individual practitioner in treatment success [47] it cannot be ruled out that the results obtained in the study stemmed from a non-specific effect of the experimental treatment.

Last, the study conducted by Castro-Sànchez et al. [45] was designed to compare the effects of craniosacral therapy (CST) with massage on disability, pain intensity, quality of life, and mobility in patients with low back pain. One primary outcome (score obtained in the Roland Morris Disability Questionnaire) and 16 secondary outcomes were proposed. Statistical analysis made immediately after the treatment failed to demonstrate a significant difference for the primary outcome but six of the 16 secondary outcomes were found different in favor of the CST. One month later, statistical analysis demonstrated that three of the secondary outcomes were still significant in favor of CST. We should point out here that the authors tried to avoid biases but, considering the absence of effect on the primary outcome and that method induced inequity in terms of treatment duration (50 minutes for CST vs 30 minutes for massage) we cannot consider that these results contribute to the body of evidence for the specific efficacy of CST.

As a whole, our study reports that almost all studies dealing with reliability or efficacy of cranial osteopathy were determined to have a high risk of bias. At the same time we note that these biases (particularly lack of a control group, lack of blinding of the examiners and inappropriate statistical analysis) would lead to an artificial increase in reliability or treatment effects. As a consequence, we have to interpret results in favor of cranial osteopathy with caution when lack of reliability or treatment effect is a strong argument to consider the technique as scientifically unfounded.

Within this context, we would like to provide guidance on generating high quality evidence in the field of cranial osteopathy. First, we note that many items in studies included in our review were rated as having an unclear risk of bias. This point could be solved if authors pay close attention to giving a detailed description of the methods they used. However, we appreciate that many scientific journals limit the length of the articles. Authors often choose to shorten the methods section, reducing thus the opportunity the reader to identify potential bias. We recommend publishing articles in journals with no restriction regarding the article length.

For the studies of diagnosis reliability in cranial osteopathy, naturally we recommend that future researchers to use the items proposed in our study and inspired from QAREL. We must be particularly vigilant about the personal expertise of the examiners and avoid those whose training is not fully completed. We should add that the tool we proposed was designed to specifically assess the risk of bias linked to the study methods but that reliability was not evaluated, representing one of the limitations of our study. For inter-rater reliability studies, as much as possible must be done to ensure that exchange of information between examiners is not possible during the tests. Thus, procedures extending over several days are not recommended. This point leads us to consider strategies to avoid memorization of the results by the examiners. First, the order of assessments (subjects and examiners) has to be randomized and no information about subjects, outside of that necessary for the examination, should be communicated to the examiners. In addition, blinding of subjects and examiners has to be as strict as possible. On this last point, Halma et al. [32] proposed a quite outstanding plan to isolate the examiner from tactile, visual, auditory and olfactory cues. Note also that for studies involving simultaneous evaluation of a subject by two separate examiners, the method sections detailed in studies by Rogers et al. [28], Moran & Gibson [30] and Sommerfeld et al. [31], should serve as models for this methodological approach.

Not surprisingly, we advise future researchers to refer to the Cochrane risk of bias tool in order to build the ideal efficacy study. However, we must mention that the reliability of this tool was only evaluated as fair for most of its items constituting another limitation of our study [51]. This tool or training in the use of this tool should be enhanced. Note that the 2010 CONSORT checklist will help significantly to design a precise randomized controlled clinical trial in the field of cranial osteopathy and we can also recommend the good methodological precaution taken by Elden et al. [42] and Haller et al. [44]. However, those two studies suffer from the confusion made between the specific and contextual effects. The main reason of this confusion is that not only do the techniques used between groups differ but also many other parameters (such as duration, practitioner etc.) differ. In order to avoid this bias, future researchers should standardized as rigorously as possible the context of the treatments proposed to the different groups in terms of number and duration of sessions, doctor-patient relationship, *etc*. Another point to mention is that in most studies, no attempt has been made to evaluate the credibility of the placebo used. This point should readily be included in future studies and could partially compensate the lack of blinding procedure inherent to the field. Last, we should underline the importance of clearly defining only one primary outcome and of avoiding multiple comparisons. If not possible, researchers should at least have planned an inflated alpha risk correction and prefer objective outcomes.

Taken together, our critical appraisal of the studies included in our review lead us to conclude that there is no evidence at present for the specific efficacy of techniques or therapeutic strategies used in cranial osteopathy. Our results are consistent with those of previous reviews on the same topic [6,9–11] and underline the need to improve methodological standards of research dealing with manual therapies in general, and osteopathy in particular.

## Conclusion

We found no evidence to support the reliability of diagnoses made using cranial osteopathy. Most existing and available studies were vulnerable to a high risk of bias and failed to demonstrate any reliability for selected outcomes. Very few well conducted trials are available demonstrating the clinical efficacy of techniques and therapeutic strategies used in cranial osteopathy. Most are seriously flawed and only two had a low risk of bias and modest results that cannot be ruled out as being due to non-specific effects of treatments. At present, there is insufficient evidence to support cranial osteopathy as being relevant for the diagnosis or treatment of patients.
